# Piecing together the mosaic of rare skin diseases: an interview with Veronica Kinsler

**DOI:** 10.1242/dmm.050636

**Published:** 2024-01-18

**Authors:** Veronica A. Kinsler

**Affiliations:** The Francis Crick Institute, 30 Guilford Street, London WC1N 3JH, UK


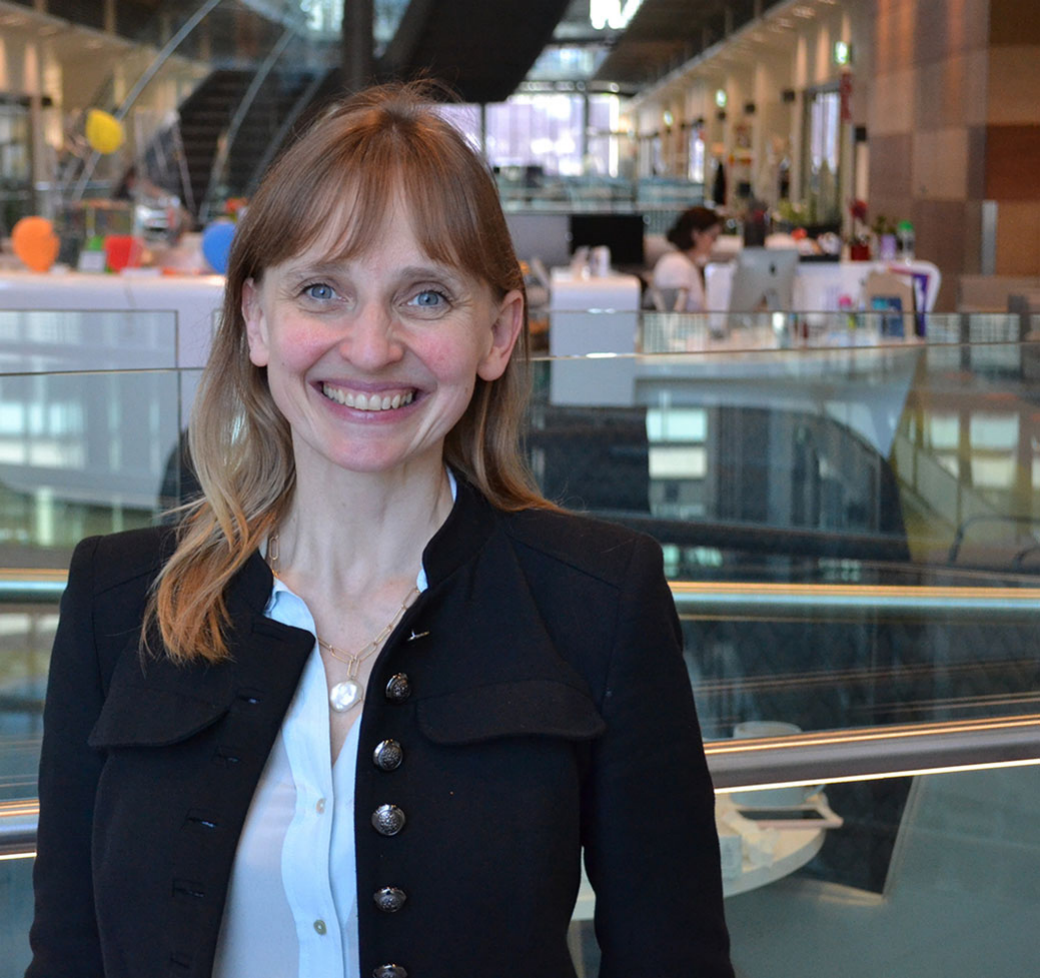


**Veronica Kinsler**

Veronica Kinsler is a clinician scientist who has dedicated her career to researching rare paediatric skin diseases. Having discovered the genetic cause for multiple mosaic skin diseases that, by definition, arise from somatic mutations during embryonic development, her ultimate goal is to translate these findings into targeted therapies. Much of her research is driven by her dedication to her patients and her partnerships with several patient advocacy groups.

Veronica initially studied medicine at the University of Cambridge, and then trained in paediatric dermatology. In parallel, she pursued research, undertaking a PhD and then a post-doctoral position in Molecular Genetics at University College London (UCL), with pump-priming funds from Caring Matters Now and Butterfly AVM Charity, and fellowships from the Wellcome Trust. She first established her research group at UCL, before being seconded to The Francis Crick Institute, London, where she is now Principal Group Leader of the Mosaicism and Precision Medicine Lab, and Assistant Research Director for Clinical Academic Training and Development. She has continued to be active in the clinic throughout her research career and is currently the Chair of Paediatric Dermatology and Dermatogenetics at Great Ormond Street Hospital (GOSH) for Children where she directs the NHS England Rare Disease Collaborative Network for Mosaic Diseases. In 2021 she was awarded a NIHR Research Professorship, the first in Dermatology. Beyond this, she holds/has held several prestigious international positions including, President and Past President of the European Society of Pediatric Dermatology 2020–2024, President of the World Congress of Pediatric Dermatology 2021, and Senior Editor of the Harper Textbook of Pediatric Dermatology. Interested in fostering knowledge and collaboration within the field of mosaic and rare diseases, she founded and directs the triannual London Mosaicism Symposium, and the annual Crick Rare Diseases Conference. In this interview, we discuss her motivations for and the challenges in researching rare disease, as well as the strides that have been taken in recent years to translate this research into precision medicine.

**You initially studied medicine and continue to work in the clinic but what made you decide to pursue laboratory research?**

Even when I was only studying medicine, I was interested in the research side. I published a couple of papers as a medical student, from an intercalated degree in neurophysiology and a laboratory research project during my elective. When I started at GOSH in 1997, I was fascinated by the diseases I was seeing in clinic and was particularly interested in the ‘why’ questions – for example why this disease had occurred, or ‘why it was always in a particular pattern. At a certain point, I was taking my questions round to different people to try to find some answers and, eventually, a senior histopathology consultant at GOSH, Prof Neil Sebire, said, ‘You know, you should do a PhD to try to work this all out’. So, I had to do a PhD to get the scientific questions answered. I then had to try to get funding to do the research, which was tricky, and had to have more time out of clinical training, which was also tricky but, eventually, I was able to start in the lab. At that point I became completely hooked because I could start to answer some of my long-term questions, and that was very exciting.

[…] we actually had something to pin the cause of the disease on, and once we knew what caused it, we could start looking at potential treatments.

**What research project have you found most exciting during your career?**

Probably finding the cause of the first of these mosaic diseases, congenital melanocytic naevus syndrome, which was published in 2013 (Kinsler et al., 2013). Mosaic diseases are sporadic, meaning they can affect any newborn, in any family, anywhere in the world. At the time I was looking for the cause, it wasn't well established how to investigate this type of disease. Patients have many different moles at birth, which can cover huge areas of the skin. Lots of different genetic variants had been described in single melanocytic naevi but the leap that I made was to think that, perhaps, one of those was in fact a mosaic variant rather than a somatic variant – in other words that it was a variant that had happened in a single cell during embryonic development and had caused all the different naevi in the same patient. So, I tested this hypothesis first of all using standard Sanger sequencing techniques on DNA from the skin lesions. I had really bad-quality DNA because it was mainly from old tissue, and there were a lot of technical issues. When I got the first set of results it didn't confirm my hypothesis – I didn't find the same variant in all the naevi from any one patient, some were positive and some were negative. So, I thought I was wrong and shelved that part of the project for nearly two years. But then I came across a small paper that described how to optimise Sanger sequencing to pick up very low levels of variants in tissue samples, and I went back and started the experiment all over again. The results that time confirmed the hypothesis. Decrypting that set of results on my own and realising that only I knew what the cause was, was amazing. And what it allowed me to do next was to set up a streamlined research project to prospectively recruit patients with other rare mosaic disorders in clinic, and to use next-generation sequencing techniques to pick up the genes. Then, we actually had something to pin the cause of the disease on, and once we knew what caused it, we could start looking at potential treatments.

**What have been the most astounding recent achievements in this field?**

Probably, at the moment, it is the gene discovery work under this mosaic diseases umbrella. Many more diseases were found to be mosaic than would have been predicted a decade ago. Interestingly, the genetic discoveries have led to a huge shift in our understanding of clinical phenotypes – we now know that lots of diseases we used to think were different entities, are actually part of a spectrum. The gene discoveries are also now starting to lead to targeted therapies.

These rare diseases have, therefore, given us important insights into common disease biology and into normal human embryology.

**What are the unique challenges with rare conditions both in the clinic and in research?**

I think the main thing in research is that it's hard to get funding. So much of the success of medical research depends on funding and we're competing with common diseases, which have greater socioeconomic impact and greater attraction for pharmaceutical companies. This has, however, changed somewhat recently, which is encouraging. The other issue is the number of patients. Very frequently journal reviewers want more patient samples. In practice, however, we have to ask for research biopsies and primary cultures from the skin of babies and small children, so the samples are, understandably, really limited and really precious. In most of my studies, there is no possibility of going back to double the number of samples.

A major benefit is that discoveries in the rare disease space often lead to conclusions or hypotheses about mechanisms in more common diseases. Many of the genes that we found are cancer genes, such as *NRAS* or *BRAF*. In patients with mosaic diseases, however, they only have this single-gene variant that creates a very pure model of the effects of these known oncogenes in humans. This can inform their role in the context of multiple other variants in a cancer. It's also very interesting developmentally because mosaic diseases are caused *in utero* by mutations in a single cell, so sometimes it permits visualisation of the developmental distribution of that particular cell lineage. These rare diseases have, therefore, given us important insights into common disease biology and into normal human embryology.

It has been a real partnership with the patient support grups. We couldn't do the research without them, so we definitely feel like we're working together to do this.

**You work closely with several patient groups. Why is this so important for your research?**

The reason I still do this job is largely because, every week, I go to clinic and there are new babies coming in and their parents say, ‘Why did this happen to us and what can you do about it?’. Nowadays, we can usually tell them why it happened, at least in principal if not the exact gene, but we still can't do anything about the diseases in terms of treatment. This is a really tough conversation to have with people, particularly, since it happens every week. So, I am definitely inspired and motivated by meeting patients and families, and I think our research gives them hope. We have a more than 95% participation in research from our clinics, which is incredible. Almost everyone we ask wants to take part, even though we tell them it's for research and, although we're trying to find treatments, we can't guarantee that we will succeed.

I think it has been a real partnership with the patient support groups. We couldn't do the research without them, so we definitely feel like we're working together to do this. All the researchers in the lab, even the ones that don't go to clinic, go to the patient days to give talks and meet the patients. It makes it immediate for them, and I think it helps the patient groups by giving them a degree of hope and a feeling that things are happening. So, it's definitely a two-way process.

**You set up the Great Ormond Street Hospital Rare Dermatology Diseases Resource tissue bank. Why are resources like this so important in rare disease research?**

Generally, large biobanks don't have the tissue we need. For mosaic diseases, most of our patients have normal blood and many normal tissues but, in their affected tissue, they'll have a mutation. Without that specific tissue, you can't find the cause. You also need to know how to preserve and extract DNA, RNA or protein from the relevant tissue. We are dealing with tiny tissue samples from children, and we have to make that last as long as we can. We couldn't get it from anywhere else.

We try to do a lot of thinking before we do experiments, which may sound obvious, but with a rare disease and limited funding, we have to have a good hit rate for what we do.

**You recently published two studies that suggest targeting calcium signalling would be beneficial in patients with mosaic variants in GNAQ or GNA11. How do you know when research like this has translational potential and could be taken to clinical trials?**

We try to do a lot of thinking before we do experiments, which may sound obvious, but with a rare disease and limited funding, we have to have a good hit rate for what we do. I meet patients every week and I feel that I have to stay focused on trying to find treatments for them. Pretty much everything we do has been focused on this pathway, from finding the cause of the disease, figuring out the biology and then identifying a therapy.

In those recent papers, we designed siRNA therapy to target the causative variant known to cause Sturge–Weber syndrome and the other diseases on the GNAQ/GNA11 mosaic spectrum (Knöpfel et al., 2023; Zecchin et al., 2023). There's going to be a couple of other papers on that type of therapy for other mosaic diseases from our lab in the coming year or two. I am in the process of setting up a clinical trial for the one with which we are furthest along. If we can get proof of concept for one disease, it should make funding and planning easier for the others.

For me, this is a life-long project. I started working on this in about 1998 and I'm trying to get it done before I retire! It's more of a mission than anything else.

**How do you balance clinical work and your fundamental research, and how do you create symbiosis between the two roles?**

It is tricky to get the balance right and, actually, it's ultimately dependent on funding. If you don't get research funding, then your role in the NHS becomes all-consuming and many clinicians who would like to do research really struggle to get any research done. If, however, you can get research funding, then you are able to reduce the amount of time in clinical work. I do one day a week in the NHS and then four days a week in the lab. You need that time if you're doing basic research to make any head way. That's the sort of balance that's ideal for me. The symbiosis comes from researching the diseases we see in our patients. The patients participate in the research and our research is focused on bringing things back to the patients. I also get my best ideas from looking at the patients and their skin conditions, so I feel very fortunate to be able to do both.

**What do you enjoy doing outside of the lab and work?**

For many years, I worked part time and I also had six years where I worked extremely part-time – just a half a day or one day a week – when I was looking after my children. So, I think in every previous spare moment outside the lab I was picking up the children from school, doing homework and making meals, very intensively for many years. So, there wasn't much time for a lot of other things. About two years ago, my youngest child went to university, so that's changed, and I feel like I have more time outside of work now. What I really like is dancing. I've always done some sort of dancing. I used to do a lot of ballet when I was young, then I did flamenco and, more recently, tango.

**Did you find coming back to work after your career break challenging?**

I never fully stopped, although I was extremely part time for ages, and I think that helped. It maintains your confidence to some degree, and it keeps your hand in with changes in the workplace. But coming back into a PhD on a 50% timetable was tough. Like many other ‘part-time’ people I was really doing 50% time so that I could have flexibility, and in practice was doing a lot of additional hours, often in the evening or at weekends. In some ways it was great, I was really happy to be back at work because I was so excited about the research. But in other ways it was really tough. If you've had time out for children you have to work twice as hard as anybody else, mainly because people don't take you seriously. You have to be better than the best.

[…] for goodness' sake, if we are potentially clever enough to solve fundamental biological problems and cure diseases, I am pretty sure we can sort out how to get flexibility into the workplace.

I try to support people that are trying to combine work with other areas of their lives. Although this affects women and men, the main problem remains with retaining women in science, not so much in medicine these days. Many leaders in science are still labouring under the misapprehension that you can't do science part time. Actually, what you need is the option for a large amount of flexibility for a long time and, for goodness’ sake, if we are potentially clever enough to solve fundamental biological problems and cure diseases, I am pretty sure we can sort out how to get flexibility into the workplace.

I used to try to hide those parts of my CV because they were big holes but, at a certain point, I realised it was important and I started putting it on my CV, and when I moved to the Crick, I put my part-time career on my public facing website. Since then, many people have contacted me asking how it went because they want to do the same.
